# Crystal structure of 3-acetyl-4*H*-chromen-4-one

**DOI:** 10.1107/S2056989015012098

**Published:** 2015-06-30

**Authors:** Yoshinobu Ishikawa

**Affiliations:** a School of Pharmaceutical Sciences, University of Shizuoka, 52-1 Yada, Suruga-ku, Shizuoka 422-8526, Japan

**Keywords:** crystal structure, chromone, hydrogen bond, π–π stacking

## Abstract

In the title compound, C_11_H_8_O_3_, the fused-ring system is almost planar (r.m.s. deviation = 0.020 Å), with the largest deviation from the least-squares plane [0.0462 (17) Å] being for a pyran C atom. The dihedral angle between the plane of the fused-ring system and acetyl plane is 5.149 (16)°. In the crystal, the fused rings are linked by aromatic π–π stacking inter­actions [centroid–centroid distance between the benzene and pyran rings = 3.643 (6) Å] and C—H⋯O hydrogen bonds, generating a three-dimensional network.

## Related literature   

For a related structure, see: Chanda *et al.* (2014 ▸). For further synthetic details, see: Yokoe *et al.* (1994 ▸); Li *et al.* (2012 ▸).
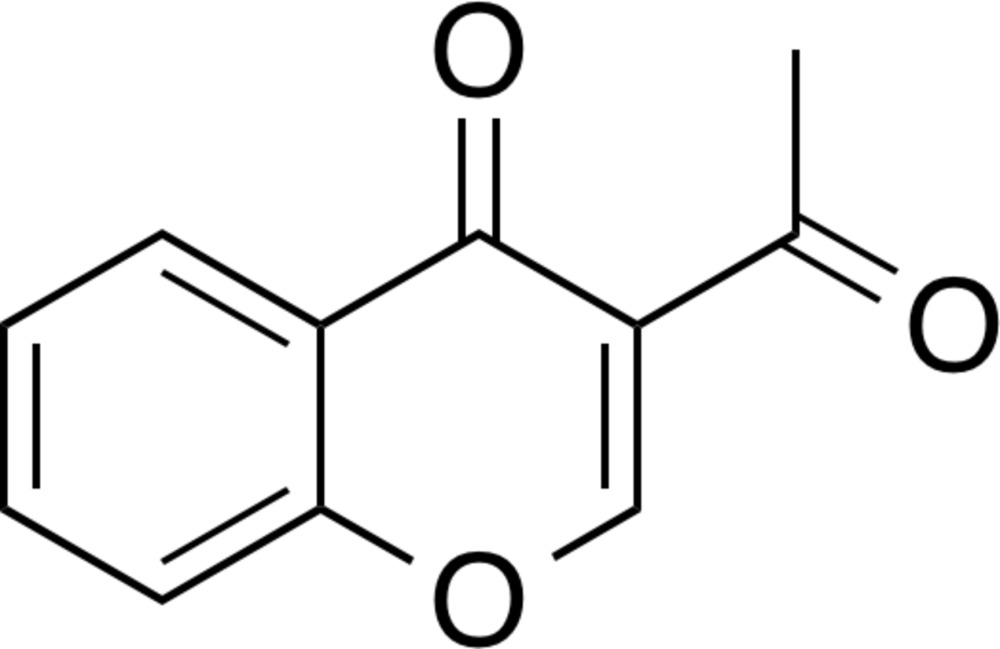


## Experimental   

### Crystal data  

C_11_H_8_O_3_
*M* *_r_* = 188.18
Monoclinic, 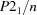

*a* = 8.016 (13) Å
*b* = 25.93 (6) Å
*c* = 4.091 (8) Å
β = 94.79 (14)°
*V* = 847 (3) Å^3^
*Z* = 4
Mo *K*α radiation
μ = 0.11 mm^−1^
*T* = 100 K
0.42 × 0.25 × 0.20 mm

### Data collection  

Rigaku AFC-7R diffractometer
2377 measured reflections
1962 independent reflections
1510 reflections with *F* ^2^ > 2.0σ(*F* ^2^)
*R*_int_ = 0.018
3 standard reflections every 150 reflections intensity decay: −0.5%

### Refinement  

*R*[*F* ^2^ > 2σ(*F* ^2^)] = 0.041
*wR*(*F* ^2^) = 0.112
*S* = 1.03
1959 reflections
128 parameters
H-atom parameters constrained
Δρ_max_ = 0.31 e Å^−3^
Δρ_min_ = −0.20 e Å^−3^

### 

Data collection: *WinAFC Diffractometer Control Software* (Rigaku, 1999 ▸); cell refinement: *WinAFC Diffractometer Control Software*; data reduction: *WinAFC Diffractometer Control Software*; program(s) used to solve structure: *SIR2008* (Burla, *et al.*, 2007 ▸); program(s) used to refine structure: *SHELXL97* (Sheldrick, 2008 ▸); molecular graphics: *CrystalStructure* (Rigaku, 2010 ▸); software used to prepare material for publication: *CrystalStructure*.

## Supplementary Material

Crystal structure: contains datablock(s) General, I. DOI: 10.1107/S2056989015012098/hb7454sup1.cif

Structure factors: contains datablock(s) I. DOI: 10.1107/S2056989015012098/hb7454Isup2.hkl

Supporting information file. DOI: 10.1107/S2056989015012098/hb7454Isup3.cml

**Fig. 1.**
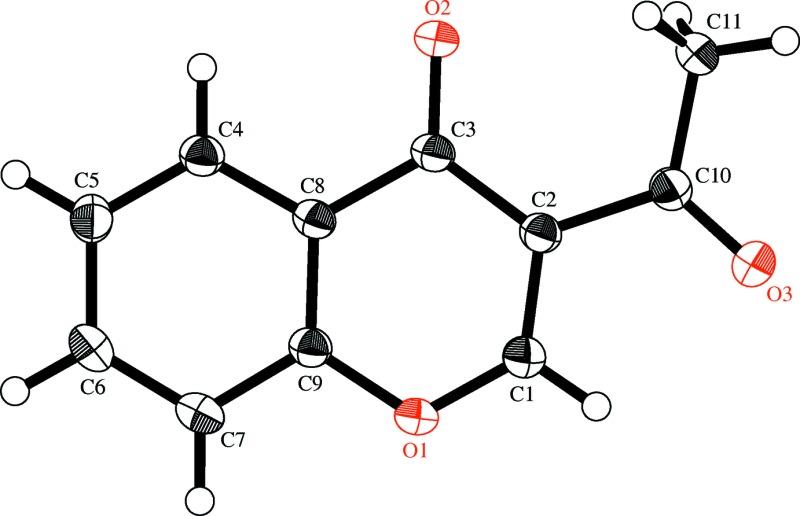
The molecular structure of the title compound, with displacement ellipsoids drawn at the 50% probability level. Hydrogen atoms are shown as small spheres of arbitrary radius.

. DOI: 10.1107/S2056989015012098/hb7454fig1.tif
The mol­ecular structure of the title compound, with displacement ellipsoids drawn at the 50% probability level. Hydrogen atoms are shown as small spheres of arbitrary radius.

**Fig. 2.**
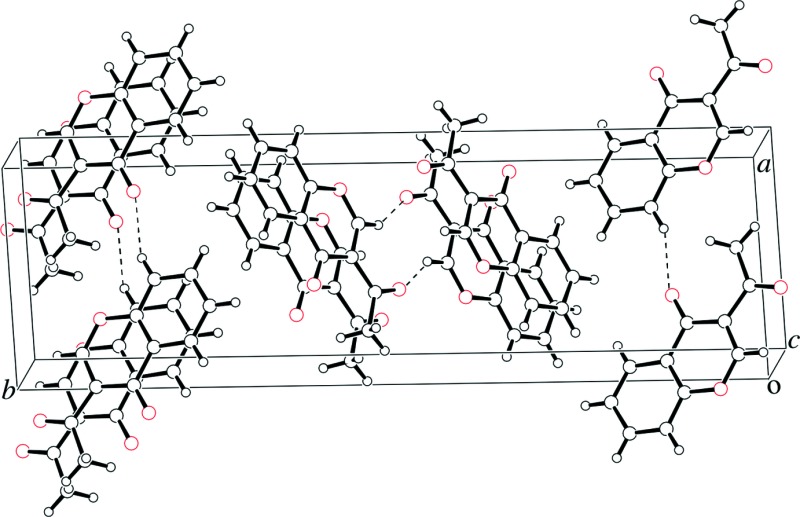
A view of the intermolecular interactions of the title compound. C–H···O hydrogen bonds are represented as dashed lines.

**Fig. 3.**
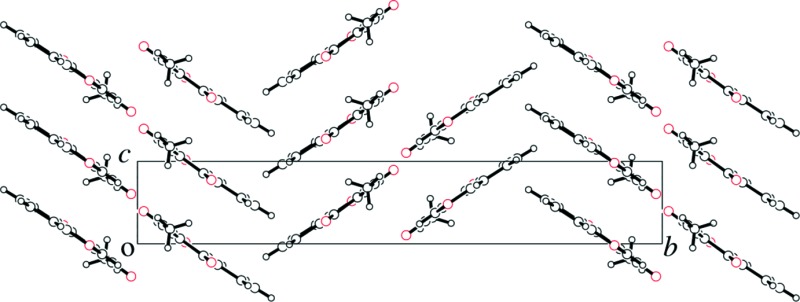
A view of the title compound down to the a-axis.

. DOI: 10.1107/S2056989015012098/hb7454fig2.tif
A view of the inter­molecular inter­actions of the title compound. C–H⋯O hydrogen bonds are represented as dashed lines.

a . DOI: 10.1107/S2056989015012098/hb7454fig3.tif
A view of the title compound down to the *a*-axis.

CCDC reference: 1408496

Additional supporting information:  crystallographic information; 3D view; checkCIF report

## Figures and Tables

**Table 1:** Hydrogen-bond geometry (, )

*D*H*A*	*D*H	H*A*	*D* *A*	*D*H*A*
C7H5O2^i^	0.95	2.40	3.292(6)	155
C1H1O3^ii^	0.95	2.31	3.264(5)	148

Symmetry codes: (i) 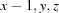; (ii) 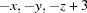.

## supplementary crystallographic information

### S1. Comment

Many derivatives of the title compound are reported because of their chemical, biological and medicinal significance (Yokoe *et al.* 1994, Chanda *et al.* 2014).

The mean deviation of the least-square plane for the non-hydrogen atoms of the fused-ring is 0.0201 Å, and the largest deviation from the plane is 0.0462 (17) Å for C2. These mean that these atoms are essentially coplanar (Fig.1). The dihedral angle between the fused-ring and acetyl plane is 5.149 (16) Å.

In the crystal, the molecules are linked by π–π stacking [centroid–centroid distance between the benzene and pyran rings = 3.643 (6) Å], and C–H···O hydrogen bonds form sheets along [0 4 1] and [0 4 1], as shown in Fig.2 and Fig.3.

The crystal structure of a 2,5,6,7-substituted 3-acetylchromone derivative is reported (Chanda *et al.* 2014).

### S2. Experimental

The title compound was synthesized from 3-(dimethylamino)-1-(2-hydroxyphenyl)prop-2-enone (Li *et al.* 2012) according to the literature method (Yokoe *et al.* 1994). Single crystals suitable for X-ray diffraction were obtained by slow evaporation of an ethyl acetate solution of the title compound at room temperature.

### S3. Refinement

All hydrogen atoms were placed in geometrical positions [C–H 0.95 and 0.98 Å], and refined using a riding model with *U*_iso_(H) = 1.2*U*_eq_ of the parent atoms. The s.u.s for the cell parameters are rather large, possibly due to frost damage to the crystal.

### Crystal data

**Table:**

C_11_H_8_O_3_	*F*(000) = 392.00
*M**_r_* = 188.18	*D*_x_ = 1.475 Mg m^−^^3^
Monoclinic, *P*2_1_/*n*	Mo *K*α radiation, λ = 0.71069 Å
Hall symbol: -P 2yn	Cell parameters from 25 reflections
*a* = 8.016 (13) Å	θ = 15.2–17.5°
*b* = 25.93 (6) Å	µ = 0.11 mm^−^^1^
*c* = 4.091 (8) Å	*T* = 100 K
β = 94.79 (14)°	Prismatic, colorless
*V* = 847 (3) Å^3^	0.42 × 0.25 × 0.20 mm
*Z* = 4	

### Data collection

**Table:**

Rigaku AFC-7R diffractometer	θ_max_ = 27.6°
ω scans	*h* = −5→10
2377 measured reflections	*k* = 0→33
1962 independent reflections	*l* = −5→5
1510 reflections with *F*^2^ > 2.0σ(*F*^2^)	3 standard reflections every 150 reflections
*R*_int_ = 0.018	intensity decay: −0.5%

### Refinement

**Table:**

Refinement on *F*^2^	Secondary atom site location: difference Fourier map
*R*[*F*^2^ > 2σ(*F*^2^)] = 0.041	Hydrogen site location: inferred from neighbouring sites
*wR*(*F*^2^) = 0.112	H-atom parameters constrained
*S* = 1.03	*w* = 1/[σ^2^(*F*_o_^2^) + (0.0509*P*)^2^ + 0.3687*P*] where *P* = (*F*_o_^2^ + 2*F*_c_^2^)/3
1959 reflections	(Δ/σ)_max_ < 0.001
128 parameters	Δρ_max_ = 0.31 e Å^−^^3^
0 restraints	Δρ_min_ = −0.20 e Å^−^^3^
Primary atom site location: structure-invariant direct methods	

### Special details

**Table:**

Refinement. Refinement was performed using all reflections. The weighted *R*-factor (*wR*) and goodness of fit (*S*) are based on *F*^2^. *R*-factor (gt) are based on *F*. The threshold expression of *F*^2^ > 2.0 σ(*F*^2^) is used only for calculating *R*-factor (gt).

### Fractional atomic coordinates and isotropic or equivalent isotropic displacement parameters (Å^2^)

**Table:**

	*x*	*y*	*z*	*U*_iso_*/*U*_eq_	
O1	−0.18103 (13)	0.09136 (4)	1.0184 (3)	0.0256 (3)	
O2	0.27801 (13)	0.14078 (4)	0.7749 (3)	0.0296 (3)	
O3	0.22177 (14)	0.01208 (4)	1.3956 (4)	0.0352 (3)	
C1	−0.03986 (18)	0.06711 (6)	1.1256 (4)	0.0239 (4)	
C2	0.11774 (18)	0.08077 (5)	1.0636 (4)	0.0212 (3)	
C3	0.14176 (18)	0.12556 (6)	0.8560 (4)	0.0210 (3)	
C4	−0.01216 (18)	0.19777 (6)	0.5570 (4)	0.0233 (4)	
C5	−0.1584 (2)	0.22372 (6)	0.4646 (4)	0.0264 (4)	
C6	−0.31159 (19)	0.20407 (6)	0.5507 (4)	0.0268 (4)	
C7	−0.31830 (18)	0.15967 (6)	0.7318 (4)	0.0251 (4)	
C8	−0.01438 (17)	0.15278 (5)	0.7452 (4)	0.0202 (3)	
C9	−0.16842 (18)	0.13482 (5)	0.8296 (4)	0.0212 (3)	
C10	0.25597 (19)	0.04850 (6)	1.2256 (4)	0.0232 (4)	
C11	0.43401 (19)	0.06180 (6)	1.1822 (5)	0.0270 (4)	
H1	−0.0510	0.0374	1.2581	0.0286*	
H2	0.0912	0.2105	0.4924	0.0280*	
H3	−0.1554	0.2549	0.3429	0.0316*	
H4	−0.4124	0.2217	0.4826	0.0322*	
H5	−0.4223	0.1463	0.7889	0.0301*	
H6A	0.5082	0.0387	1.3165	0.0324*	
H7B	0.4557	0.0976	1.2506	0.0324*	
H8C	0.4550	0.0579	0.9509	0.0324*	

### Atomic displacement parameters (Å^2^)

**Table:**

	*U*^11^	*U*^22^	*U*^33^	*U*^12^	*U*^13^	*U*^23^
O1	0.0172 (5)	0.0250 (6)	0.0351 (6)	−0.0015 (4)	0.0041 (5)	0.0048 (5)
O2	0.0169 (6)	0.0302 (6)	0.0419 (7)	−0.0011 (5)	0.0043 (5)	0.0109 (5)
O3	0.0269 (6)	0.0319 (7)	0.0468 (8)	0.0004 (5)	0.0032 (6)	0.0164 (6)
C1	0.0211 (7)	0.0222 (7)	0.0282 (8)	−0.0004 (6)	0.0018 (6)	0.0013 (6)
C2	0.0183 (7)	0.0202 (7)	0.0250 (8)	−0.0003 (6)	0.0018 (6)	−0.0014 (6)
C3	0.0175 (7)	0.0213 (7)	0.0243 (8)	−0.0013 (6)	0.0026 (6)	−0.0023 (6)
C4	0.0207 (7)	0.0231 (7)	0.0261 (8)	−0.0014 (6)	0.0015 (6)	−0.0012 (6)
C5	0.0273 (8)	0.0234 (8)	0.0280 (8)	0.0021 (6)	−0.0001 (7)	0.0017 (7)
C6	0.0205 (8)	0.0310 (9)	0.0285 (8)	0.0056 (6)	0.0000 (6)	−0.0010 (7)
C7	0.0178 (7)	0.0293 (8)	0.0283 (8)	0.0001 (6)	0.0028 (6)	−0.0031 (7)
C8	0.0175 (7)	0.0204 (7)	0.0227 (8)	−0.0003 (6)	0.0017 (6)	−0.0036 (6)
C9	0.0192 (7)	0.0206 (7)	0.0239 (8)	−0.0007 (6)	0.0023 (6)	−0.0020 (6)
C10	0.0217 (8)	0.0224 (7)	0.0253 (8)	0.0008 (6)	0.0017 (6)	0.0001 (6)
C11	0.0193 (8)	0.0288 (8)	0.0327 (9)	0.0018 (6)	0.0011 (6)	0.0060 (7)

### Geometric parameters (Å, º)

**Table:**

O1—C1	1.336 (3)	C7—C9	1.392 (3)
O1—C9	1.375 (3)	C8—C9	1.390 (3)
O2—C3	1.233 (3)	C10—C11	1.493 (3)
O3—C10	1.218 (3)	C1—H1	0.950
C1—C2	1.356 (3)	C4—H2	0.950
C2—C3	1.461 (3)	C5—H3	0.950
C2—C10	1.498 (3)	C6—H4	0.950
C3—C8	1.475 (3)	C7—H5	0.950
C4—C5	1.377 (3)	C11—H6A	0.980
C4—C8	1.399 (3)	C11—H7B	0.980
C5—C6	1.401 (3)	C11—H8C	0.980
C6—C7	1.373 (4)		
			
C1—O1—C9	118.05 (15)	O3—C10—C11	120.67 (16)
O1—C1—C2	126.32 (18)	C2—C10—C11	119.82 (17)
C1—C2—C3	119.10 (15)	O1—C1—H1	116.840
C1—C2—C10	115.94 (17)	C2—C1—H1	116.843
C3—C2—C10	124.94 (16)	C5—C4—H2	119.697
O2—C3—C2	125.01 (15)	C8—C4—H2	119.697
O2—C3—C8	120.81 (18)	C4—C5—H3	120.092
C2—C3—C8	114.17 (16)	C6—C5—H3	120.097
C5—C4—C8	120.61 (17)	C5—C6—H4	119.500
C4—C5—C6	119.81 (19)	C7—C6—H4	119.487
C5—C6—C7	121.01 (16)	C6—C7—H5	120.923
C6—C7—C9	118.14 (17)	C9—C7—H5	120.937
C3—C8—C4	121.26 (16)	C10—C11—H6A	109.476
C3—C8—C9	120.79 (17)	C10—C11—H7B	109.464
C4—C8—C9	117.95 (15)	C10—C11—H8C	109.473
O1—C9—C7	116.03 (16)	H6A—C11—H7B	109.469
O1—C9—C8	121.52 (15)	H6A—C11—H8C	109.475
C7—C9—C8	122.45 (17)	H7B—C11—H8C	109.471
O3—C10—C2	119.50 (17)		
			
C1—O1—C9—C7	−178.13 (12)	H2—C4—C5—H3	−2.1
C1—O1—C9—C8	1.35 (19)	H2—C4—C8—C3	2.2
C9—O1—C1—C2	−0.8 (3)	H2—C4—C8—C9	−178.8
C9—O1—C1—H1	179.2	C4—C5—C6—C7	1.3 (3)
O1—C1—C2—C3	−1.2 (3)	C4—C5—C6—H4	−178.7
O1—C1—C2—C10	177.31 (13)	H3—C5—C6—C7	−178.7
H1—C1—C2—C3	178.9	H3—C5—C6—H4	1.3
H1—C1—C2—C10	−2.7	C5—C6—C7—C9	0.4 (3)
C1—C2—C3—O2	−177.82 (14)	C5—C6—C7—H5	−179.6
C1—C2—C3—C8	2.4 (2)	H4—C6—C7—C9	−179.6
C1—C2—C10—O3	1.2 (2)	H4—C6—C7—H5	0.4
C1—C2—C10—C11	−177.68 (13)	C6—C7—C9—O1	178.20 (13)
C3—C2—C10—O3	179.57 (13)	C6—C7—C9—C8	−1.3 (3)
C3—C2—C10—C11	0.7 (3)	H5—C7—C9—O1	−1.8
C10—C2—C3—O2	3.9 (3)	H5—C7—C9—C8	178.7
C10—C2—C3—C8	−175.94 (12)	C3—C8—C9—O1	0.0 (2)
O2—C3—C8—C4	−2.7 (3)	C3—C8—C9—C7	179.50 (12)
O2—C3—C8—C9	178.31 (13)	C4—C8—C9—O1	−178.94 (12)
C2—C3—C8—C4	177.09 (12)	C4—C8—C9—C7	0.5 (2)
C2—C3—C8—C9	−1.87 (19)	O3—C10—C11—H6A	−3.2
C5—C4—C8—C3	−177.81 (13)	O3—C10—C11—H7B	−123.2
C5—C4—C8—C9	1.2 (2)	O3—C10—C11—H8C	116.8
C8—C4—C5—C6	−2.0 (3)	C2—C10—C11—H6A	175.7
C8—C4—C5—H3	177.9	C2—C10—C11—H7B	55.7
H2—C4—C5—C6	178.0	C2—C10—C11—H8C	−64.3

### Hydrogen-bond geometry (Å, º)

**Table:**

*D*—H···*A*	*D*—H	H···*A*	*D*···*A*	*D*—H···*A*
C7—H5···O2^i^	0.95	2.40	3.292 (6)	155
C1—H1···O3^ii^	0.95	2.31	3.264 (5)	148

Symmetry codes: (i) *x*−1, *y*, *z*; (ii) −*x*, −*y*, −*z*+3.
