# Supplementary figures and images for: Crystal structure of 3-acetyl-4H-chromen-4-one
Source: Acta Crystallogr E Crystallogr Commun. 2015 Jun 30;71(Pt 7):o527. doi: 10.1107/S2056989015012098 (PMC4518922; doi:10.1107/S2056989015012098)

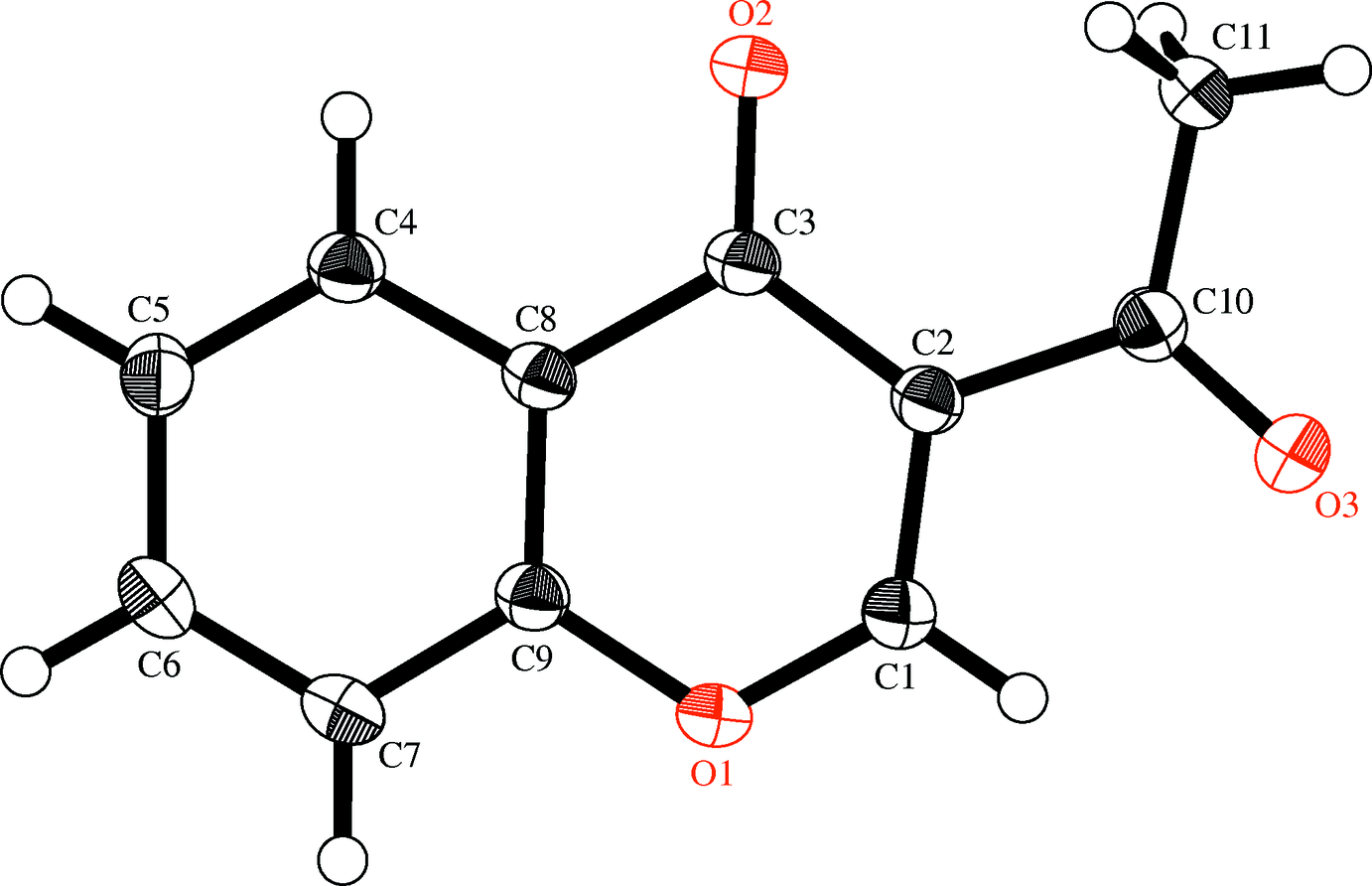

Supplement: Supplementary file 4 [file e-71-0o527-fig1.tif]

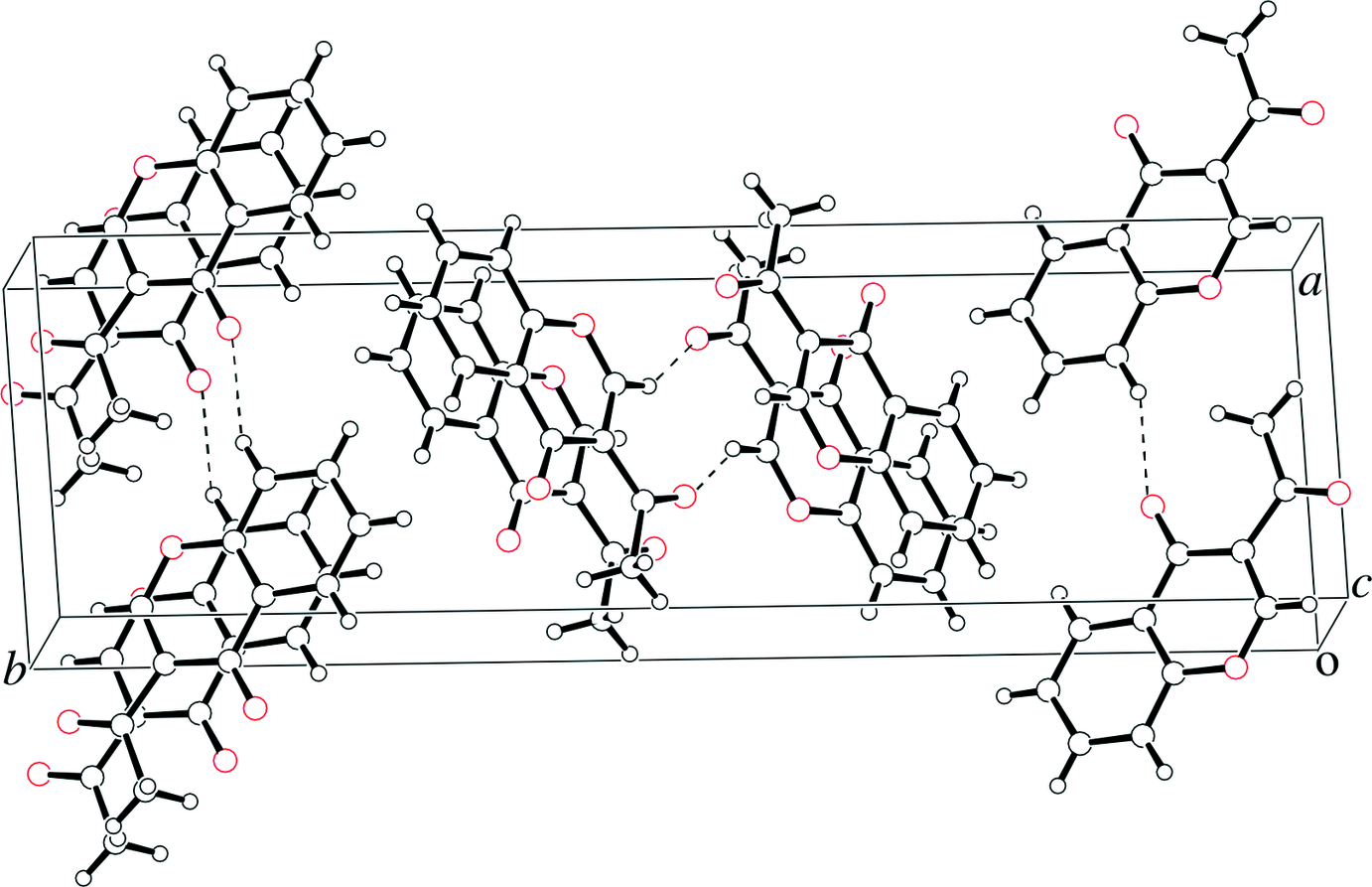

Supplement: Supplementary file 5 [file e-71-0o527-fig2.tif]

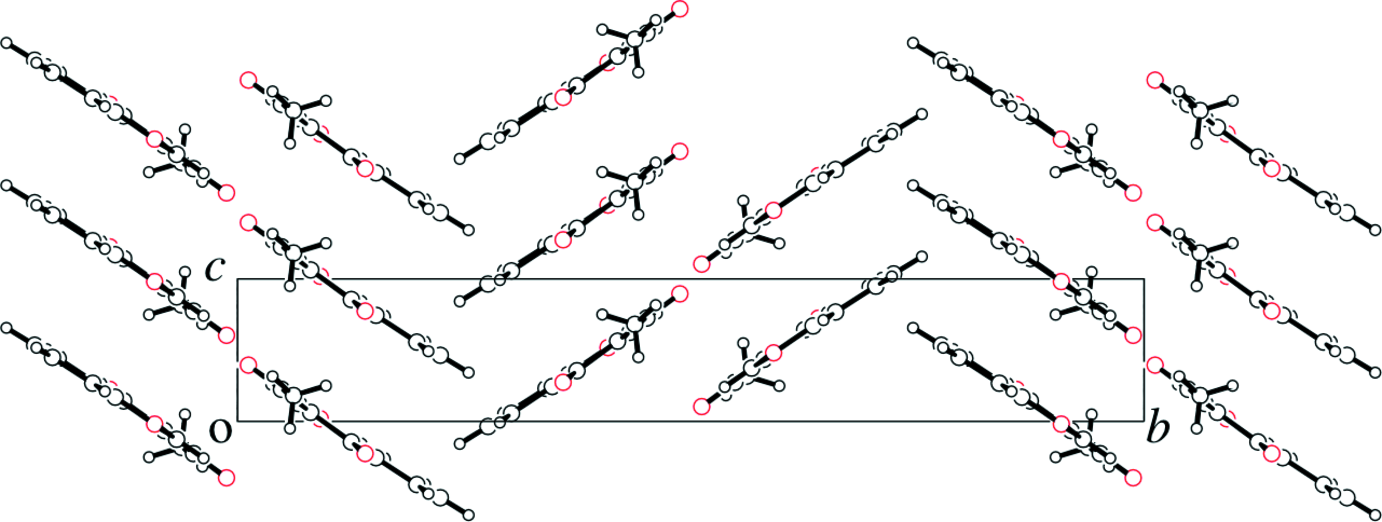

Supplement: Supplementary file 6 [file e-71-0o527-fig3.tif]
